# Ultralight Carbon Aerogels with Highly Hierarchical Porous Structures Synthesized from Sodium Alginate-Nanocellulose Composites for High-Performance Supercapacitors

**DOI:** 10.3390/polym17111544

**Published:** 2025-06-01

**Authors:** Jinran Cui, Yexin Dai, Shuo Xu, Pingping Zhang, Zhiyun Wang, Xianhua Liu

**Affiliations:** 1 School of Environmental Science and Engineering, Tianjin University, Tianjin 300354, China; jinrancui@tju.edu.cn (J.C.); daiyexinf@163.com (Y.D.); xushuo001226@163.com (S.X.); 2 College of Food Science and Engineering, Tianjin Agricultural University, Tianjin 300384, China; zpp@tjau.edu.cn

**Keywords:** nano-cellulose, sodium alginate, carbon aerogel, sustainable energy

## Abstract

Hierarchical porous carbon materials hold great potential for energy storage applications due to their high porosity, large specific surface area, and excellent electrical conductivity. Cellulose and sodium alginate are naturally abundant high-molecular-weight biopolymer materials. Utilizing them as precursors for the fabrication of high-performance electrochemical carbon materials is highly significant for achieving carbon neutrality goals. In this study, porous carbon aerogels were successfully synthesized using a combination of freeze-drying and a simple carbonization process, with nanocellulose and sodium alginate as precursors. Among the prepared samples, SC-0.03 (sodium alginate: nanocellulose = 0.1:0.03) exhibited the best performance, achieving a specific surface area of 713.7 m^2^ g^−1^. This material features an optimized hierarchical pore structure and a substantial intrinsic oxygen doping content, resulting in excellent capacitance performance. Benefiting from these structural advantages and their synergistic effects, the SC-0.03 electrode demonstrated a high specific capacitance of 251.5 F g^−1^ at a current density of 0.5 A g^−1^. This study shows that the construction of three-dimensional porous structures by taking advantage of the self-supporting properties of natural polymer materials does not require the introduction of external binders. Due to the nanoscale dimensions and high aspect ratio, nanocellulose enables the formation of a more refined and interconnected hierarchical pore network, enhancing ion accessibility and conductivity. The hierarchical porous carbon aerogel developed in this study, based on a biomass self-reinforcement strategy, not only shows great promise as an advanced energy storage material but also possesses environmentally friendly properties, offering new insights for the development of sustainable energy materials.

## 1. Introduction

Supercapacitors have emerged as a key focus in next-generation energy storage systems due to their exceptional environmental compatibility, broad operating temperature range, ultra-long cycle life, and millisecond-scale charge–discharge rate [1]. Notably, carbon-based composites, with their unique electronic transport networks, physical stability, and intrinsic safety, have dominated electrochemical interface energy storage. In particular, their role in double-layer charge storage mechanisms is irreplaceable [2]. However, the surface charge adsorption principle that governs their energy storage limits their energy density, restricting industrial applications in high-end fields such as power supply systems [3]. To overcome this limitation, recent research has achieved significant advancements in the structural engineering of carbon-based electrodes, optimizing multiple performance parameters through precise multi-dimensional pore system design: (i) The controlled distribution of high-specific-surface-area active sites significantly enhances charge storage capacity [4]; (ii) Nano-topological engineering creates a three-dimensional conductive network that shortens ion diffusion paths and reduces interfacial impedance [5,6]; (iii) A hierarchical pore system—integrating micro-, meso-, and macropores—ensures efficient electrolyte infiltration while establishing rapid charge transfer channels [7,8]; (iv) Surface chemical reconfiguration, achieved through lattice defect engineering and heteroatom doping, enhances pseudocapacitive contributions and precisely regulates interfacial charge distribution [9,10]. The synergistic effect of these multi-component energy storage mechanisms, enabled by cross-scale structural design and chemical modification, is driving supercapacitors toward a breakthrough in high-energy-density electrochemical energy storage devices [11].

As a prominent representative of third-generation porous carbon materials, carbon aerogel has become a core electrode material for high-power-density supercapacitors due to its ultra-low packing density, three-dimensional interconnected topology, highly tunable pore structure, and excellent electron mobility [12]. However, the conventional preparation method relies on phenol-formaldehyde polycondensation and requires complex processes such as sol–gel self-assembly, solvent exchange, supercritical fluid drying, and high-temperature pyrolysis. These challenges, including high process complexity, poor batch stability, and organic solvent residues, significantly hinder its industrial application [13]. A promising alternative is the green synthesis of carbon aerogels from biomass-derived precursors such as chitin polysaccharides and bacterial cellulose. These natural polymers offer renewability, structural diversity, and inherent element doping, while the derived carbon aerogels exhibit gradient pore distribution (micro-, meso-, and macropores) and intrinsic chemical modifications [14]. At the energy storage level, the hierarchical pore structure enables functional differentiation: micropores (<2 nm) form charge storage interfaces, mesopores (2–50 nm) facilitate electrolyte transport, and macropores (>50 nm) serve as ion reservoirs [15]. Moreover, the intrinsic heteroatom enrichment (e.g., N, O, S) in biomass precursors enables surface polarity tuning, improving electrode–electrolyte compatibility (reducing contact angle by 40–60%) and enhancing charge storage through pseudocapacitive effects (30–45% contribution) [16]. These modifications also reconstruct electron transport channels, improving conductivity by 2–3 orders of magnitude. For instance, Yu et al. synthesized N, O co-doped carbon aerogel/polypyrrole composites from cattail (*Typha orientalis*), achieving a binder-free supercapacitor with a maximum surface capacitance of 419 mF cm^−2^ at 1 mA cm^−2^ and 86.4% capacitance retention after 3000 charge–discharge cycles [17]. Similarly, Phan Minh Tu et al. developed iron-doped magnetic carbon aerogels from palm shells, attaining a high specific capacitance of 203 F g^−1^ at 50 mV s^−1^ with stable performance over 500 cycles [18]. Zhao et al. prepared carbon composite materials based on corn stalks and sodium alginate (SA). It has a multi-level porous structure, an excellent specific surface area, and outstanding specific capacitance and cycling stability, which has promoted the development of high-performance supercapacitors [19]. Yazan Al Haj et al. regulated the properties of nanocellucellulose aerogel through conjugated anions, maintained the structural integrity after carbonization, and significantly improved the electrochemical performance [20]. Combined with electrode wetting technology, it provided a new path for the development of sustainable supercapacitors. Other carbon materials also have new applications in supercapacitors. Shaik Junied Arbaz et al. proposed a simple synthesis of nickel-molybdenum oxide nanoparticles injected into biocarbon microfibers (NiMoO NPs@BCMFs) for energy storage, derived from the fungus *Laetiporus sulfureus* [21]. This material exhibited an area capacity of 113 µAh cm^−2^ in the three-electrode system and maintained a retention rate of 104% after 20,000 cycles. Combining the negative electrode BCMFS/NF, a biofriendly micro supercapacitor (BMSC) was constructed, with an energy density of 56 µWh cm^−2^ and a power density of 11,250 µW cm^−2^, showing a good application prospect. As a multifunctional material, two-dimensional graphite carbon nitride is used in electrochemical energy storage applications due to its nitrogen-rich adsorption sites, cost-effective production and adjustable electronic structure [22]. Asmaa M. Elsayed et al. prepared β-Ni(OH)_2_/graphite carbon nitride (G-C_3_N_4_) nanocomposites and brain-shaped polypyrrole by hydrothermal polymerization and oxidative polymerization, respectively. The specific electricity values of the supercapacitors without and with polypyrrole are 101 and 120 F/g, respectively. Furthermore, after the addition of polypyrrole, the retention stability increased from 85.6% to 90.2% up to 500 cycles [23]. Bai et al. prepared hierarchical porous activated carbons from a SA/bacterial cellulose composite via carbonization and KOH activation [24]. The resulting materials exhibit a 3D interconnected porous structure, high graphitization degree, and rich oxygen-containing functional groups, leading to excellent electrochemical performance with a specific capacitance of 302 F/g and strong cycling stability. These findings suggest that natural biopolymers can be effectively used to develop sustainable and high-performance electrode materials for supercapacitors.

Despite these advancements, several challenges remain in the development of biomass-derived carbon aerogels for electrochemical energy storage [25]. First, existing biomass-based carbon aerogels often suffer from insufficient electrical conductivity and poor structural integrity, limiting their rate performance and long-term stability [26]. Second, while heteroatom doping has been explored, achieving precise control over the doping concentration and distribution to maximize electrochemical activity remains a challenge [27]. Additionally, most current studies focus on single biomass sources, whereas the synergistic combination of multiple biomass precursors with complementary properties has not been fully explored [28]. Moreover, the large-scale, green synthesis of carbon aerogels with well-defined hierarchical pore structures and tailored surface chemistry is still in its early stages, requiring further optimization to balance electrochemical performance with sustainability [29].

To address these challenges, this study aims to synthesize carbon aerogels with highly hierarchical porous structures using SA and nanocellulose through a straightforward freeze-drying and carbonization process. Although SA is commonly used in the food and cosmetics industries, it offers several advantages that make it highly suitable for carbon aerogel preparation. These include its excellent gelation properties, high content of functional groups (carboxyl and hydroxyl) that facilitate cross-linking and carbonization, and its ability to form stable three-dimensional porous networks through self-assembly and freeze-drying. Moreover, SA has been demonstrated in previous studies to yield carbon materials with superior textural and electrochemical properties. While alternatives such as sodium carboxymethylcellulose are indeed abundant, they often require additional cross-linking agents or structure-directing steps, making SA a more straightforward and efficient precursor for fabricating highly porous carbon aerogels with minimal processing complexity. This study produced high-performance composite carbon aerogel from two biomass materials, representing the successful implementation of an organic solvent-free, streamlined, and environmentally friendly manufacturing process—signifying a major breakthrough in green material fabrication. Compared to traditional methods, the new approach not only simplifies production and eliminates the use of toxic solvents but also significantly reduces environmental impact, offering a novel and practical pathway for developing high-performance, sustainable materials. The resulting aerogel exhibits a high specific surface area, an optimized hierarchical pore structure (coordinating micropores, mesopores, and macropores), and substantial intrinsic oxygen doping, all contributing to its excellent capacitance and rate performance. This approach not only boosts electrochemical performance but also supports sustainable development goals, underscoring its potential in advancing eco-friendly energy storage technologies.

## 2. Materials and Methods

### 2.1. Materials

Nanocellulose was purchased from Naxiansi New Material Co. (Zhongshan, China). Sodium alginate was supplied by Xiensi Opod Technology Co., Ltd. (Tianjin, China); Polytetrafluoroethylene dispersion (60%) and carbon black were purchased from Yilongsheng Co. (Suzhou, China). All the materials were used without any further purification.

### 2.2. Preparation of Composite Carbon Aerogel

**Figure 1:**
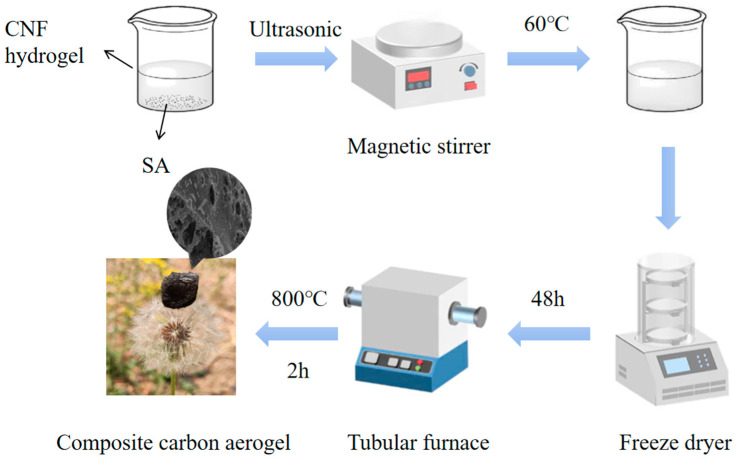
Flow chart of composite carbon aerogel preparation.

First, prepare a 2.5 wt% nanocellulose (CNF) hydrogel and a 1 wt% sodium alginate (SA) solution. To prepare the SA solution, dissolve 1 g of sodium alginate in 99 g of deionized water. Subject the mixture to ultrasonic treatment in an ultrasonic cleaner set at 100% frequency for 4–5 h until a uniform dispersion is achieved. Next, prepare four 100 mL beakers. Add 10 g of the 1 wt% sodium alginate solution to each beaker. Then, add 0 g, 0.4 g, 1.2 g, and 2.0 g of the 2.5 wt% CNF hydrogel to the beakers, respectively, and label them as SC-0, SC-0.01, SC-0.03, and SC-0.05. Adjust the total mass of each mixture to 20 g by adding an appropriate amount of distilled water. Place the beakers on a rotor mixer and stir at 60 °C for 6 h to ensure uniform mixing of sodium alginate and nanocellulose. After mixing, transfer each solution to a 5 mL glass container and allow it to stand for 1 h at room temperature to stabilize. Then, freeze the samples in the lower compartment of a refrigerator for 6 h until fully solidified. Once frozen, transfer the samples to a freeze dryer and subject them to freeze-drying for 48 h to obtain the aerogels. Finally, remove the dried aerogels from the glass containers and place them in a porcelain boat. Transfer the boat to a tube furnace and heat the samples at a rate of 5 °C/min to 800 °C. Maintain this temperature for 2 h for carbonization, then allow the furnace to cool naturally to room temperature. The resulting carbon aerogels are shown in Figure 1.

### 2.3. Electrode Preparation

First, dilute the PTFE dispersion with distilled water to a concentration of 5 wt%. Cut the nickel foam into 1 cm × 1 cm squares. Grind the sample material into a fine powder, then mix it with carbon black and the diluted PTFE dispersion in a mass ratio of 8:1:1. The loading amount of the active substance is 8 mg. During mixing, add a small amount of ethanol to promote dispersion. The resulting homogeneous slurry is then uniformly coated onto a 1 cm^2^ piece of nickel foam and dried in an oven at 80 °C for 12 h. Next, place another piece of nickel foam of the same dimensions over the coated sample to form a sandwich structure of “nickel foam-carbon material-nickel foam”. This assembly is pressed using a tablet press at 15 MPa for 30 s to obtain the final electrode sheet. The prepared electrode is mounted onto an electrode holder and integrated into a three-electrode system, using a platinum sheet as the counter electrode and a mercury/mercury oxide (Hg/HgO) electrode as the reference.

### 2.4. Characterization

Scanning electron microscopy (SEM, S-4800, Hitachi, Japan) was used to observe the surface morphology of the samples. The functional groups present in the composite carbon aerogel were identified using Fourier transform infrared (FTIR) spectroscopy. The crystal structure of the carbon aerogel samples was analyzed using X-ray diffraction (XRD). X-ray photoelectron spectroscopy (XPS) was employed to determine the core-level binding energies of elements such as C1s and O1s, and detailed peak fitting analysis was conducted to investigate their chemical states. The material’s wettability was evaluated through contact angle measurements. Thermogravimetric analysis (TGA) was performed to assess the thermal stability and retention of material properties at elevated temperatures. Nitrogen adsorption–desorption isotherms were obtained to determine the specific surface area and pore volume, and to analyze the pore size distribution of the samples. The compressive properties of the aerogel samples were measured using a universal testing machine. Cyclic voltammetry (CV), galvanostatic charge–discharge (GCD), and electrochemical impedance spectroscopy (EIS) were evaluated in 2 mol/L KOH solutions.

## 3. Results and Discussion

### 3.1. Micromorphology Characterization

**Figure 2:**
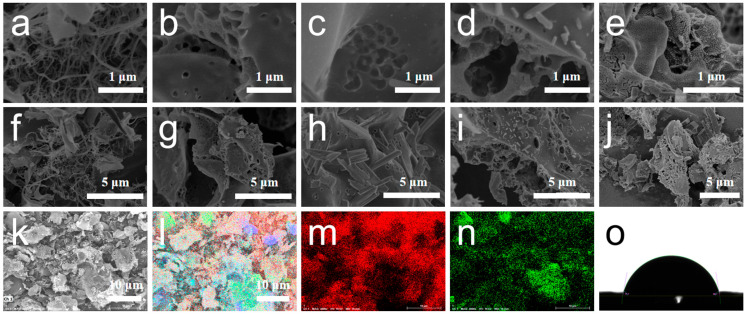
Electron microscopic images of CNF (**a**), SA (**b**), SC-0.01 (**c**), SC-0.03 (**d**), and SC-0.05 (**e**) with 1 μm scale bar; Electron microscopic images of CNF (**f**), SA (**g**), SC-0.01 (**h**), SC-0.03 (**i**), and SC-0.05 (**j**) with 5 μm scale bar; (**k**–**n**) EDS elemental distribution maps of C, O in SC-0.03; (**o**) Contact angle image of SC-0.03.

Figure 2a–j present the SEM images of carbon aerogels prepared from pure nanocellulose, pure sodium alginate, and composites SC-0.01, SC-0.03, and SC-0.05. The carbon aerogel derived from pure nanocellulose exhibits an irregular, filamentous structure formed by the entanglement of fibrous networks. In contrast, the carbon aerogel derived from pure sodium alginate (SA) displays a bulk-like morphology with a well-defined porous surface. The composite carbon aerogels (SC-X) exhibit structural features intermediate between the fibrous and block-like forms, suggesting a hybrid morphology. This distinct structure is attributed to both physical interactions between nanocellulose and sodium alginate, as well as chemical bonding arising from the reaction between their functional groups [30,31]. At low doping levels of nanocellulose, the overall morphology remains largely unchanged, with the addition of rod-like structures appearing on the surface. As the nanocellulose content increases, these rod-like structures become more refined and uniformly distributed, resulting in a denser structure and significantly enhanced overall porosity. This abundant porous architecture facilitates faster electron and ion transport during electrochemical reactions, thereby improving electrochemical performance. However, excessive nanocellulose addition can lead to surface “dry cracking” and hinder pore formation. This may be due to the high concentration filling in the originally formed pores, which ultimately impairs electrochemical transport. These observations indicate that nanocellulose plays a crucial role in modulating the morphology and structure of sodium alginate-based carbon aerogels, contributing to enhanced electrochemical performance when used in appropriate amounts.

Figure 2k–n show the EDS elemental mapping of carbon (C) and oxygen (O) in the SC-0.03 sample. The results indicate that both elements are uniformly distributed throughout the material [32]. This uniform elemental distribution can be attributed to the high carbon and oxygen content in the raw materials—nanocellulose and sodium alginate. After carbonization, some carbon-rich impurities also become fused into the matrix and remain in the structure. Additionally, since all electrochemical measurements in this study were conducted in aqueous electrolyte, the hydrophilicity of the materials plays a critical role in determining their performance. Good hydrophilicity ensures that the material surface is adequately wetted by the electrolyte, providing a favorable interface for electrolyte ion adsorption during the charge–discharge process and promoting efficient ion transport at the electrode–electrolyte interface. In this study, the water contact angle of the SC-0.03 sample was measured to assess its hydrophilicity. The contact angles of the sample surface were 79.7° (Figure 2o), indicating a certain degree of hydrophilicity [33]. Strong hydrophilicity enables rapid wetting of the material’s surface, allowing for more effective utilization of its surface area and internal structure, thereby enhancing resource utilization efficiency. The hydrophilicity of carbon materials is primarily governed by the presence of surface functional groups. At elevated activation temperatures, heteroatoms such as oxygen and hydrogen are significantly removed from the material’s surface, resulting in a reduction in hydrophilic functional groups. This, in turn, hinders the ability of the surface to be effectively wetted by the electrolyte, potentially compromising electrochemical performance.

### 3.2. Physical and Chemical Structure Characterization

**Figure 3:**
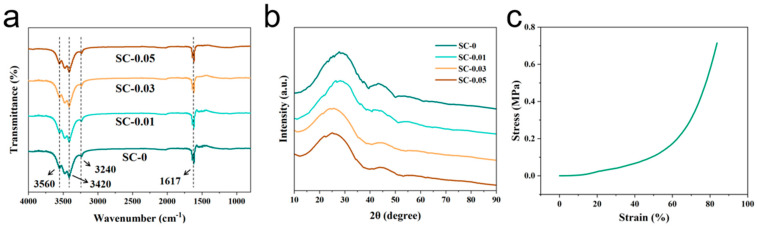
(**a**) FT-IR spectra, (**b**) XRD pattern, and (**c**) stress–strain diagram of compressive mechanical properties of SC-0.03.

To further analyze the variation in functional groups with different proportions of raw materials, fourier transform infrared (FTIR) spectroscopy was conducted on pure sodium alginate (SA) carbon aerogel (used as a blank control, SC-0) and carbon aerogels SC-0.01, SC-0.03, and SC-0.05, each containing increasing amounts of nanocellulose (Figure 3a). The broad absorption bands observed at 3420 cm^−1^ and 3560 cm^−1^ in the SC samples correspond to the stretching vibrations of –OH groups, indicating both intramolecular and intermolecular hydrogen bonding [34]. The peak at 3240 cm^−1^ also corresponds to –OH stretching, suggesting that sodium alginate and nanocellulose interact to form a polyhydroxy polymer during the composite formation process. Furthermore, the absorption peak at 1617 cm^−1^ is attributed to the stretching vibration of C=O. Notably, prominent –OH and C=O stretching vibration peaks appeared in all four spectra, which is related to the natural presence of glucuronic acid units in SA and the presence of hydroxyl groups in CNF. Some of these functional groups are involved in crosslinking during the formation of the composite aerogel [35].

The crystallographic structure of SC-X was characterized by X-ray diffraction (XRD), as shown in Figure 3b. The diffraction patterns of SC-0, SC-0.01, SC-0.03, and SC-0.05 exhibit similar trends, with prominent characteristic peaks observed at 2θ ≈ 24° and 43°, corresponding to the (002) and (100) planes of graphite, respectively [36]. These broad peaks suggest a low degree of graphitization in all samples. Notably, no additional characteristic peaks from other elements are present in the XRD patterns, indicating that the majority of the non-carbon components—such as sodium from sodium alginate and nanocellulose—were effectively removed during high-temperature carbonization, yielding predominantly pure carbon materials. The (002) diffraction peak for SC-0 appears at 23.95°. With increasing nanocellulose content, this peak shifts progressively to lower angles: 24.73° for SC-0.05, 26.46° for SC-0.03, and 27.66° for SC-0.01. This leftward shift suggests that higher nanocellulose concentrations lead to greater gas evolution—primarily CO_2_ and CO—during pyrolysis. The escape of these gases etches more carbon from the carbonized structure, introducing additional defects and increasing the interlayer spacing within the carbon matrix. Consequently, this results in a more disordered carbon structure with a higher degree of structural defects.

The SC-0.03 composite carbon aerogel also exhibit outstanding mechanical strength, as shown in Figure 3c. This excellent performance is primarily attributed to the inherent mechanical robustness of nanocellulose and the strong hydrogen bonding interactions formed between SA and CNF. Additionally, the freeze-drying process contributes to the material’s structural integrity by forming a layered architecture, which further enhances its mechanical stability. Moreover, the presence of functional groups such as carboxyl groups in nanocellulose and intermolecular van der Waals forces provide additional rigidity to the composite. The viscosity and cohesive nature of sodium alginate also help maintain the overall structural coherence of the aerogel [37]. The stress–strain behavior shown in the figure reveals that the material maintains a high level of structural integrity throughout the compression process. From 0% to 20% strain, the pressure increases only slightly, indicating minimal resistance during the initial deformation. Between 20% and 60% strain, slight fluctuations in pressure are observed, likely due to the gradual collapse and compression of the porous structure. In the final stage of compression, the layered architecture becomes denser, requiring significantly more pressure to induce further deformation. This is reflected in the steep increase in the slope of the stress–strain curve, indicating a sharp rise in pressure with increasing strain. The excellent mechanical strength and resilient porous structure suggest that the SC-0.03 carbon aerogel has great potential for practical applications, particularly in energy storage systems where mechanical durability and efficient ion transport are critical for optimizing electrochemical performance.

**Figure 4:**
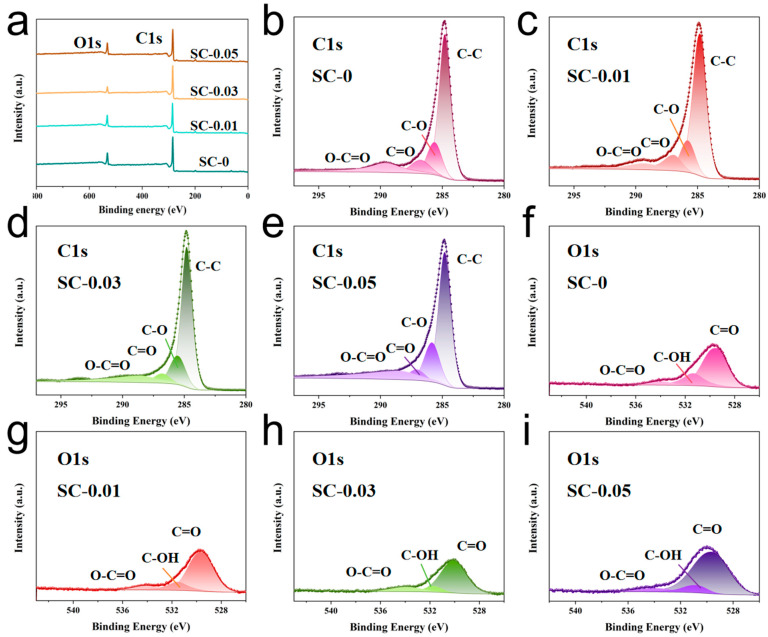
(**a**) Full spectrum of XPS elements of SC-X; (**b**–**e**) The deconvoluted high-resolution XPS peaks of SC-X samples for C1s; (**f**–**i**) The deconvoluted high-resolution XPS peaks of SC-X samples for O1s.

The elemental composition and chemical states of the SC-X samples were analyzed using X-ray photoelectron spectroscopy (XPS). After carbonization, two prominent peaks were observed at 284.8 eV and 532.2 eV, corresponding to the C1s and O1s orbitals, respectively (Figure 4a) [38]. High-resolution XPS spectra of the four samples are shown in Figure 4b–i, with the corresponding chemical bond types labeled in order of their binding energies. The C1s spectrum can be decomposed into four different peaks, located at approximately 284.8 eV, 285.8 eV, 287.0 eV and 289.6 eV, respectively, which are attributed to the C–C, C–O, C=O and O–C=O bonds, respectively [39]. Similarly, the O1s spectrum shows three peaks located at approximately 530.2 eV, 531.5 eV and 534.2 eV, corresponding to the C=O, C–OH and O–C=O functional groups, respectively [19]. These XPS results are consistent with the FTIR analysis, confirming that both nanocellulose and sodium alginate are rich in hydroxyl and carboxyl groups. The presence of these functional groups facilitates the formation of hydrogen bonds, enabling effective cross-linking within the composite structure [40]. Furthermore, the abundance of polar groups enhances the surface wettability of the carbon aerogels, which plays a critical role in improving electrolyte accessibility and, consequently, electrochemical performance.

**Figure 5:**
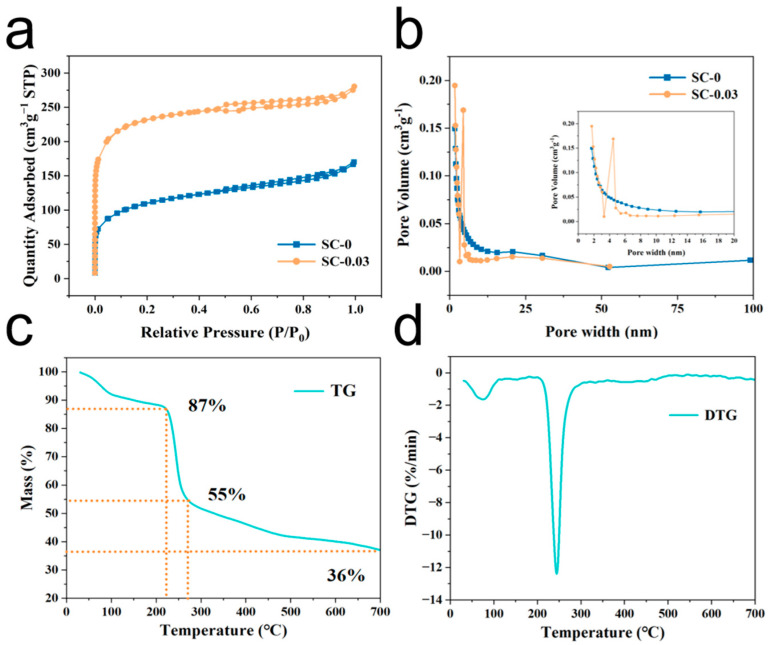
(**a**) Nitrogen absorption and desorption curve, (**b**) aperture distribution curve, (**c**) thermogravimetric curve, and (**d**) differential thermal analysis curve of SC-0.03 sample.

The specific surface area and pore structure of the SC-X samples were evaluated through nitrogen adsorption–desorption isotherms. As shown in Figure 5a, the isotherms of SC-0 and SC-0.03 display characteristics of a combined Type I and Type IV(a) isotherm. In the low-pressure region (P/P_0_ < 0.02), the curves exhibit a steep increase nearly perpendicular to the x-axis, indicating rapid adsorption due to the presence of abundant micropores. This sharp uptake suggests that the adsorbate quickly fills the micropore volume. In the mid-pressure range (P/P_0_ > 0.4), the appearance of H4-type hysteresis loops further confirms the presence of mesopores, which are primarily attributed to the crosslinked network structure of the material. Additionally, a gradual increase near the high-pressure end (P/P_0_ ≈ 1.0) indicates the existence of macropores within the structure. The corresponding pore size distribution curve (Figure 5b) supports this analysis. A significant number of micropores are observed in the 1.0–2.0 nm range [41], while mesopores are widely distributed between 2.0 and 50 nm. A small proportion of macropores, primarily under 100 nm, are also present. In summary, the SC-X composite aerogels exhibit a hierarchical porous structure comprising micropores, mesopores, and macropores. This multi-scale pore architecture is expected to facilitate efficient electrolyte penetration and ion transport, thereby enhancing electrochemical performance.

The specific surface areas of the SC-X samples were further evaluated using the Brunauer–Emmett–Teller (BET) method, with the corresponding data summarized in Table 1. The analysis reveals that SC-0 exhibits a surface area of 358.8 m^2^ g^−1^. This relatively high specific surface area is primarily attributed to the thermal decomposition of oxygen-containing functional groups in sodium alginate, which generates water vapor and carbon dioxide during high-temperature carbonization. These gaseous byproducts act as self-templates, forming pores within the carbon matrix. However, in SC-0, the majority of the pore volume consists of mesopores, while the proportions of micropores and macropores remain relatively low. Additionally, some of the micropores do not contribute effectively to energy storage due to limited ion transport pathways, which hinders their accessibility. In contrast, SC-0.03 demonstrates a marked increase in surface area, reaching 713.7 m^2^ g^−1^. This significant enhancement is attributed to the incorporation of nanocellulose, which not only introduces additional porosity but also contributes to the structural transformation of micropores into mesopores during carbonization [42]. This occurs as CO_2_ generated during pyrolysis disrupts the carbon framework, expanding pore sizes and improving connectivity. The resulting increase in mesopore volume greatly facilitates the rapid diffusion of electrolyte ions, which in turn enhances the accessibility and utilization of micropores. Overall, this optimized hierarchical porous architecture—comprising interconnected micro-, meso-, and macropores—plays a vital role in improving the electrochemical performance of the SC-X carbon aerogels.

To investigate the behavior of the SC-0.03 aerogel during high-temperature carbonization, thermogravimetric analysis (TGA) was performed in a nitrogen atmosphere, and the corresponding TG and derivative thermogravimetry (DTG) curves are shown in Figure 5c,d. The results reveal that the composite aerogel undergoes three major stages of mass loss during the heating process. In stage 1 (30–230.7 °C), a peak is observed between 50 and 100 °C, indicating a rapid increase in the heat-loss rate. This initial weight loss, accounting for approximately 13%, is primarily attributed to the evaporation of physically and chemically bound water, which is typical for hydrophilic polysaccharides that generally retain about 10–15 wt.% of bound moisture [43]. In addition, early-stage thermal reactions such as lactonization and transglycosidic bond cleavage in sodium alginate may contribute to the observed weight loss during this phase. In stage 2 (230.7–275 °C), the material experiences a rapid mass loss of around 32%. The maximum rate of weight loss occurs around 250 °C (Figure 5d). This stage is primarily driven by the cleavage reactions of sodium alginate, resulting in the formation of more stable intermediates. Simultaneously, both sodium alginate and nanocellulose are removed in the form of water vapor at high temperatures. In stage 3 (275–700 °C), a slower, steady mass loss of approximately 19% occurs. This process involves further decomposition and decarboxylation of the intermediates, leading to the release of carbon dioxide and continued carbonization of the remaining products [44]. The escape of carbon dioxide during this stage promotes the formation of porous structures, particularly mesopores, within the material.

### 3.3. Electrochemical Characterization

**Figure 6:**
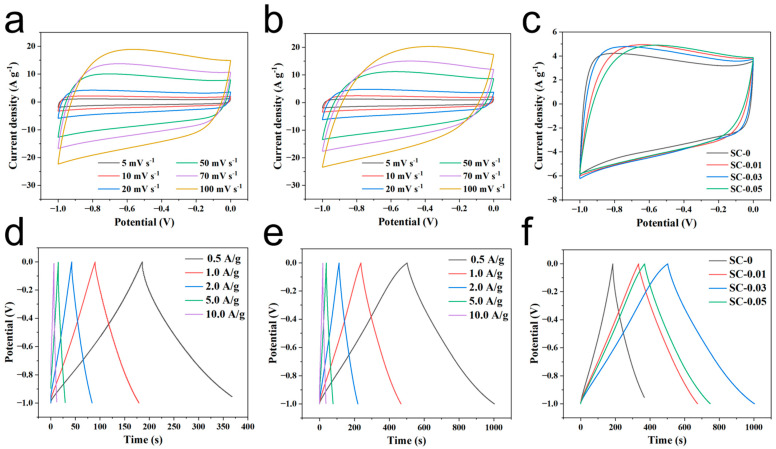
CV curves of (**a**) blank sodium alginate carbon aerogel (SC-0), and (**b**) SC-0.03 sample at different sweep speeds; (**c**) CV curves of different SC-X samples at sweep speed of 20 mV s^−1^; GCD curves of (**d**) SC-0 and (**e**) SC-0.03 at different current densities; (**f**) GCD curves of different SC-X samples at current density of 0.5 A g^−1^.

The capacitor performance of the samples was evaluated in a three-electrode supercapacitor system with 2.0 M KOH as the electrolyte [45]. As shown in Figure 6a–c, the cyclic voltammetry (CV) curve of the samples in the −1.0 to 0 V voltage window exhibits a rectangular shape, indicating that the electrode material predominantly demonstrates the capacitive behavior of double layers during charge and discharge. The small hump observed in the curve may be attributed to the weak pseudocapacitance effects caused by the oxygen atoms in sodium alginate. Among the samples, SC-0.03 exhibits the largest rectangular area on the CV curve, confirming its highest specific capacitance at the given scanning rate [46]. This enhanced performance is due to the larger specific surface area of the material, more favorable pore distribution, and optimal energy storage capabilities. Further CV tests on SC-0 and SC-0.03 at different scan rates reveal that as the scan rate increases, the area of the CV curve rectangle increases, while the corresponding specific capacitance decreases. However, the shape of the CV curve remains similar across different scan rates, which highlights the excellent reversibility of the material. This also suggests that, during rapid charge and discharge, electrolyte ions can efficiently access the active surface of the material. The observed behavior can be attributed to the well-developed hierarchical porous structure of the material.

**Figure 7:**
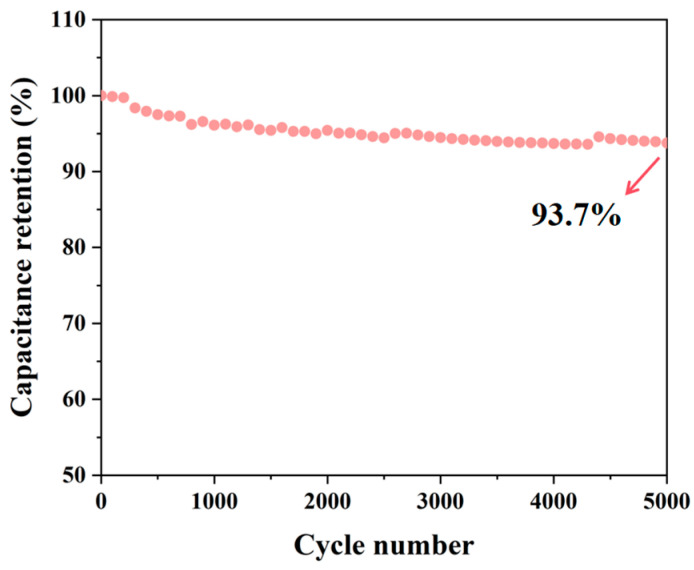
Long-cycle test of SC-0.03 at a current density of 10 A g^−1^. The red arrow indicates the final capacitance retention rate of SC-0.03.

The galvanostatic charge–discharge (GCD) curves of SC-X further support the findings from the cyclic voltammetry (CV) tests, as they display a similar pattern (Figure 6d–f). This indicates that the charge storage mechanism is dominated by double-layer adsorption on the material’s surface, confirming that the material exhibits electric double-layer capacitor behavior, with excellent coulombic efficiency and reversibility [47]. Among the samples, SC-0.03 shows the longest discharge time, which correlates with the highest specific capacitance, consistent with the CV test results. Analysis of the GCD curves reveals that the discharge time decreases with increasing current density. This is attributed to the reduced charge adsorption capacity of the material at higher current densities. The capacitance values of SC-0 and SC-0.03 at five different current densities show that SC-0.03 consistently has significantly higher capacitance. This improvement is due to the introduction of nanocellulose, which crosslinks the material into a 3D network structure. This network facilitates dynamic pore formation during high-temperature carbonization, resulting in a material with a larger specific surface area and a more developed hierarchical porous structure. SC-0.03 demonstrates the best capacitance performance, with a reversible specific capacitance of 251.5 F g^−1^ at 0.5 A g^−1^ (Figure 6f). After 5000 cycles at a current density of 10 A g^−1^, the final capacitance retention rate of SC-0.03 still reached as high as 93.7%. Moreover, it can be seen from the figure that its capacitive performance is stable with almost no huge fluctuations (Figure 7). In addition, through a comprehensive comparison of the capacitance and cycling performance of different carbon materials, the results show that the biomass composite carbon aerogel prepared in this study has good cycling stability and high specific capacitance, which is superior to the materials in previous reports (Table 2), highlighting its outstanding electrochemical energy storage capacity.

The electrochemical impedance spectroscopy (EIS) of samples SC-0, SC-0.01, SC-0.03, and SC-0.05 was evaluated using a three-electrode system to investigate their resistance characteristics. The Nyquist plots are shown in Figure S1a. In the high-frequency region, all samples exhibit a semicircular arc, where the intersection point on the x-axis represents the internal resistance (Rs), including the intrinsic resistance of the material, the contact resistance between the electrode and nickel foam, and the interfacial resistance with the electrolyte. The diameter of the semicircle reflects the charge transfer resistance (Rct); a smaller diameter indicates better conductivity and electrolyte compatibility. In the low-frequency region, the linear part of the curve represents ion diffusion behavior. A smaller slope of this line suggests slower ion diffusion within the material [55]. Among the tested samples, SC-0.03 exhibits the smallest semicircle diameter and the steepest slope in the low-frequency region, suggesting superior charge transfer kinetics and ion diffusion capabilities. The corresponding Rs values for SC-0, SC-0.01, SC-0.03, and SC-0.05 are 1.22 Ω, 1.08 Ω, 1.11 Ω, and 1.17 Ω, respectively. These results demonstrate that the introduction of CNF reduces internal impedance and enhances the conductivity of the composite materials. The improved performance of SC-0.03 can also be attributed to the hydrophilic functional groups on the CNF surface, which increase the electrolyte affinity of the carbon aerogel. However, a further increase in CNF content (as in SC-0.05) leads to a decline in the slope of the low-frequency line, indicating that excessive CNF may hinder ion transport due to structural blockage or agglomeration.

To further quantify the capacitance contribution at different scan rates, Dunn’s method was employed (Figure S1b). According to the power-law relationship *i* = a*v*^b^, where *i* is the current and *v* is the scan rate, the b-value indicates the charge storage mechanism. A b-value of 0.5 corresponds to a diffusion-controlled Faradaic process, while b = 1 implies a surface-controlled, non-diffusion-limited capacitive process. Analysis shows that the electrode material predominantly exhibits electric double-layer capacitance (EDLC) behavior at all scan rates. To distinguish between capacitive and diffusion-controlled contributions, the total current response was fitted using the equation *i*(*v*) = *k*_1_*v* + *k*_2_*v*^1/2^, where *k*_1_*v* represents the surface-controlled (EDLC) current and *k*_2_*v*^1/2^ corresponds to the diffusion-controlled pseudocapacitance. By plotting *i*/*v*^1/2^ versus *v*^1/2^, the constants *k*_1_ and *k*_2_ were extracted, allowing for the separation and quantification of each contribution. As the scan rate increased from 5 to 100 mV s^−1^, the proportion of EDLC increased from 53% to 85%, while the contribution of pseudocapacitance—mainly associated with oxygen-containing functional groups from sodium alginate—decreased from 47% to 15% (Figure S2) [56].

### 3.4. Mechanism Analysis

**Figure 8:**
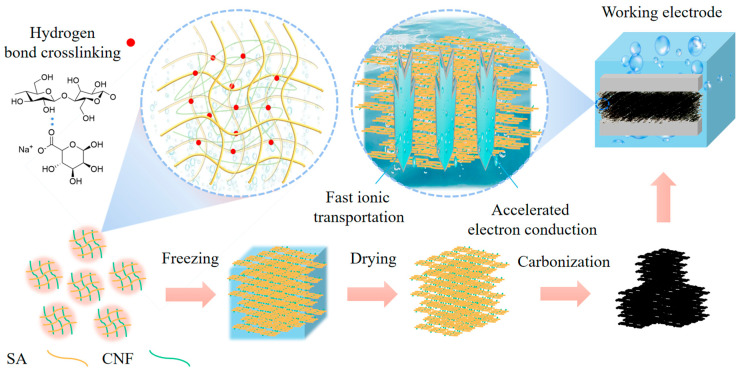
Schematic diagram of ultralight carbon aerogels preparation from sodium alginate-nanocellulose composites and working mechanisms. The red dots indicate hydrogen bonds crosslinking polymers.

The composite carbon aerogel formed from sodium alginate and nanocellulose exhibits outstanding electrochemical performance, attributed to a synergistic mechanism involving structural and chemical interactions (Figure 8). Sodium alginate and nanocellulose have established a robust three-dimensional cross-linking network through hydrogen bonds between hydroxyl groups. This hydrogen-bonded framework is preserved during freeze-drying, wherein ice crystal sublimation induces the formation of a hierarchical porous structure consisting of micropores (<2 nm), mesopores (2–50 nm), and macropores (50–100 nm). These multiscale pores provide efficient channels for rapid ion diffusion, crucial for high-rate charge–discharge processes. Subsequent carbonization at 800 °C under an inert atmosphere induces C–C bond rearrangement, forming partially graphitized microcrystalline domains that enhance electronic conductivity while preserving high porosity. The resultant carbon aerogel retains a large specific surface area (713.7 m^2^ g^–1^) and incorporates residual oxygen-containing functional groups (e.g., hydroxyl group), which contribute to pseudocapacitive charge storage via reversible redox reactions. From a performance optimization perspective, the hierarchical pore architecture and high surface area synergistically enhance ion adsorption and storage: micropores primarily contribute to EDLC, while mesopores and macropores accelerate electrolyte ion transport. Additionally, the residual oxygen-containing functional groups provide pseudo-capacitance, further enhancing the energy storage capacity. The rigid skeleton provided by nanocellulose plays a critical role in structural stability, inhibiting pore collapse during carbonization and ensuring mechanical integrity and volumetric stability during electrochemical cycling. Experimental results demonstrate that an optimal nanocellulose content of 0.03 g maximizes performance by enhancing gel strength and preserving pore architecture without causing blockage. Upon carbonization, nanocellulose-derived carbon forms a continuous conductive network, enabling efficient electron transport and coordinated ion diffusion. Furthermore, the porous carbon matrix effectively buffers volumetric expansion, providing additional durability. These findings offer a theoretical and practical foundation for the structural design and compositional tuning of high-performance energy storage devices.

## 4. Conclusions

In summary, ultralight carbon aerogels with a layered porous structure were successfully synthesized from sodium alginate–nanocellulose composites via freeze-drying and carbonization. Among the carbon aerogels prepared under varying conditions, SC-0.03 exhibited the largest pore volume and the highest specific surface area. This sample demonstrated high specific capacitance and excellent rate capability, attributed to its unique directional channel architecture, moderate C and O co-doping, and the synergistic effect of its layered pore structure. Notably, the SC-0.03 electrode delivered outstanding electrochemical performance in both 2.0 M potassium hydroxide solution and PVA/SA/KOH gel electrolyte, achieving a specific capacitance of up to 251.5 F g^−1^. This study offers a novel strategy for the practical application of biomass-derived carbon aerogels in advanced energy storage devices.

## Figures and Tables

**Table 1:** Specific surface area (m^2^ g^−1^) and pore structure (cm^3^ g^−1^) parameters of SC-X.

Samples	SBET	Smicro	Sext	Vtotal
SC-0	358.8	121.9	236.9	0.24
SC-0.03	713.7	453.5	260.2	0.42

**Table 2:** Performance comparison with previously published literature data.

Material	Current Density (A g^−1^)	Specific Capacitance (F g^−1^)	Cyclic Stability (%)	Ref.
SC-0.03	0.5	251.5	93.7	This work
SA	0.2	204	96.2	[48]
K-LWCA	0.5	201.47	70.15	[49]
Walnut shell	0.5	169.2	94.6	[50]
Graphene nanotubes	1	151.2	80.4	[51]
Lignin carbon aerogel	1	122	90	[52]
X-Cu-C	1	95.3	92.9	[53]
CAM	0.5	118	53.3	[54]

## Data Availability

The original contributions presented in this study are included in the article/supplementary material. Further inquiries can be directed to the corresponding authors.

## Supplementary Materials

The following supporting information can be downloaded at https://www.mdpi.com/article/10.3390/polym17111544/s1: Figure S1: AC impedance diagram of different SC-X samples; (b) Capacitance contribution ratio of SC-0.03 at different scan rates. Figure S2: Capacitance contribution ratio of SC-0.03 sample at different scan rates: 5 mV/s (a), 10 mV/s (b), 20 mV/s (c), 50 mV/s (d), 70 mV/s (e), and 100 mV/s (f).
