# Supplementary material for: Ultralight Carbon Aerogels with Highly Hierarchical Porous Structures Synthesized from Sodium Alginate-Nanocellulose Composites for High-Performance Supercapacitors
Source: Polymers (Basel). 2025 Jun 1;17(11):1544. doi: 10.3390/polym17111544 (PMC12158216; doi:10.3390/polym17111544)
Supplement: Supplementary file 1 [file polymers-17-01544-s001.zip › polymers-3605666-supplementary.pdf]

Supplementary Information

Ultralight carbon aerogels with highly hierarchical porous structures synthesized from sodium alginate-nanocellulose composites for high-performance supercapacitors

Jinran Cui[1], Yexin Dai[1], Shuo Xu[1], Pingping Zhang[2], Zhiyun Wang[1*],and Xianhua Liu[1*]

[1]School of Environmental Science and Engineering, Tianjin University, Tianjin, 300354, PR China;

[2]College of Food Scinece and Engineering, Tianjin Agricultural University, Tianjin, 300384, PR China;

[*]Correspondence: zhiyun_wang@tju.edu.cn(Z.W.); lxh@tju.edu.cn (X.L.)

Table of Contents

Figure S1 (a) Ac impedance diagram of different SC-X samples; (b) Capacitance contribution ratio of SC-0.03 at different scan rates.

Figure S2 Capacitance contribution ratio of SC-0.03 at different scan rates: 5 mV/s (a), 10mV/s (b), 20 mV/s (c), 50 mV/s (d), 70 mV/s (e), and 100 mV/s (f).

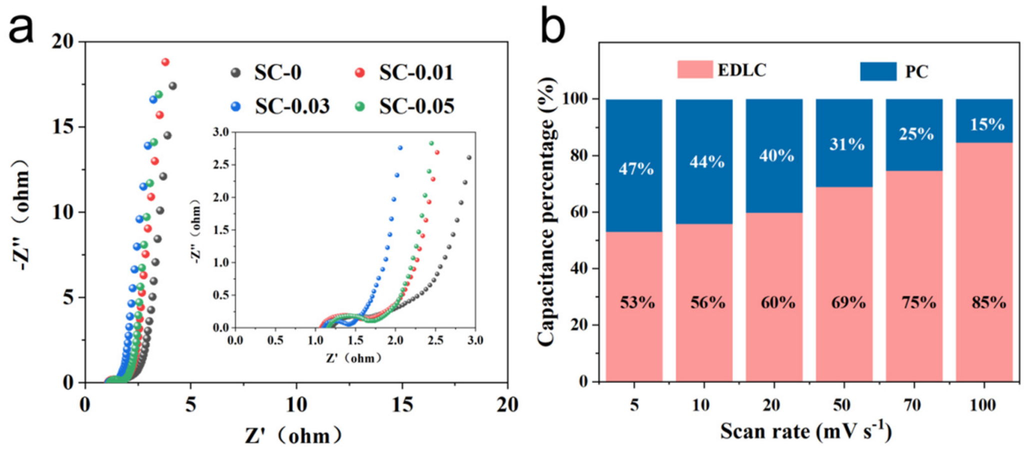

Figure S1 (a) AC impedance diagram of different SC-X samples; (b) Capacitance contribution ratio of SC-0.03 at different scan rates.

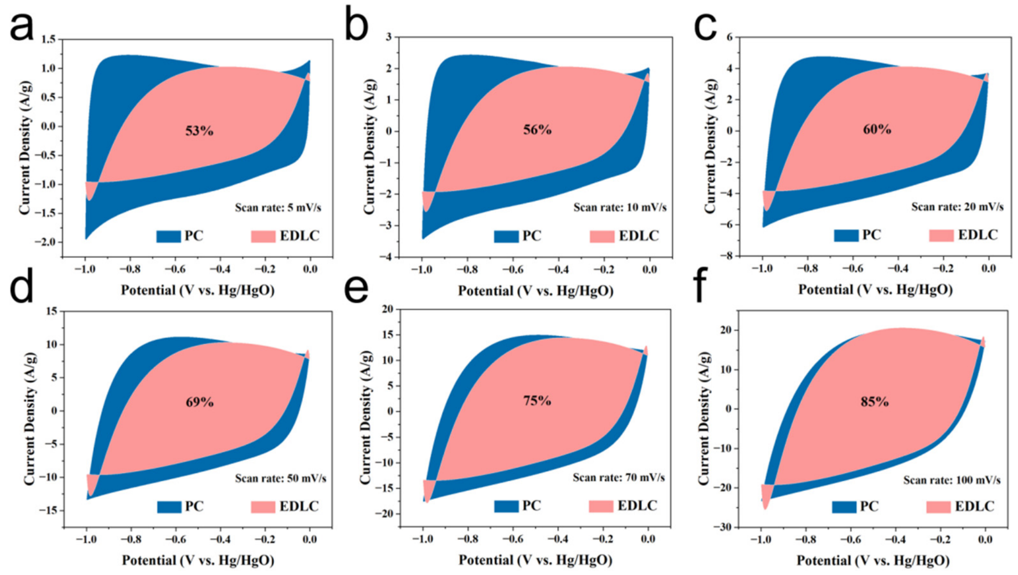

Figure S2 Capacitance contribution ratio of SC-0.03 at different scan rates: 5 mV/s (a), 10mV/s (b), 20 mV/s (c), 50 mV/s (d), 70 mV/s (e), and 100 mV/s (f).
